# Microbiome - a (FUTURE) marker for the differential diagnosis for autism spectrum disorder and attention-deficit/hyperactivity disorder?

**DOI:** 10.1192/j.eurpsy.2021.546

**Published:** 2021-08-13

**Authors:** I. Ramos, M. Figueiredo-Braga

**Affiliations:** 1 Psichology, Faculty of Medicine - University of Porto, Porto, Portugal; 2 Clinical Neurosciences And Mental Health, Faculty of Medicine, University of Porto, Porto, Portugal; 3 Metabolism, Nutrition & Endocrinology, i3S - Instituto de Investigação e Inovação em Saúde, Porto, Portugal

**Keywords:** Attention Deficit/Hyperactivity Disorder, Microbiome, autism, Biomaker

## Abstract

**Introduction:**

The differential diagnosis between Autism Spectrum Disorder (ASD) and Attention Deficit/Hyperactivity Disorder (ADHD) is often challenging and detrimental to early and timely treatment. Co-current and overlapping symptoms contribute to erode differential diagnostic accuracy, based mainly on clinical assessment supported by standardized instruments and reports from parents and teachers. The microbiota was recently considered a valuable resource in the search for biological markers in neurodevelopmental disorders.

**Objectives:**

Our objective was to examine the published literature in order to clarify the role of the microbiome as a possible differential biomarker between ASD and ADHD.

**Methods:**

Five hundred and sixteen articles were reviewed in order to contextualize the role of Gut- Brain Axis in neurodevelopment and neurodevelopmental disorders, the microbiome as a biomarker and ultimately to unravel microbiome abnormalities reported in patients diagnosed with ASD and/or ADHD.

**Results:**

Although gut microbiome appears to be involved in the pathogenesis of ASD with several reports identifying changes in gut populations and functions, a “microbial signature” is still not reached. In ADHD patients, research confirms that the composition and predicted functions of gut microbiome are also altered, but identically controversial results were found.

**Conclusions:**

Future studies are needed to confirm the relationship between the composition and function of the microbiome and the occurrence or presentation of each of the disorders. A specific signature of the microbiota could then constitute itself as a differential biomarker in ASD and ADHD.

